# Geometric Abstract Art and Public Health Data

**DOI:** 10.3201/eid2210.AC2210

**Published:** 2016-10

**Authors:** Salaam Semaan

**Affiliations:** Centers for Disease Control and Prevention, Atlanta, Georgia, USA

**Keywords:** art science connection, emerging infectious diseases, art and medicine, about the cover, infectious diseases, geometric abstract art and public health data, Piet Mondrian, Composition in White, Red, and Yellow, public health

**Figure:**
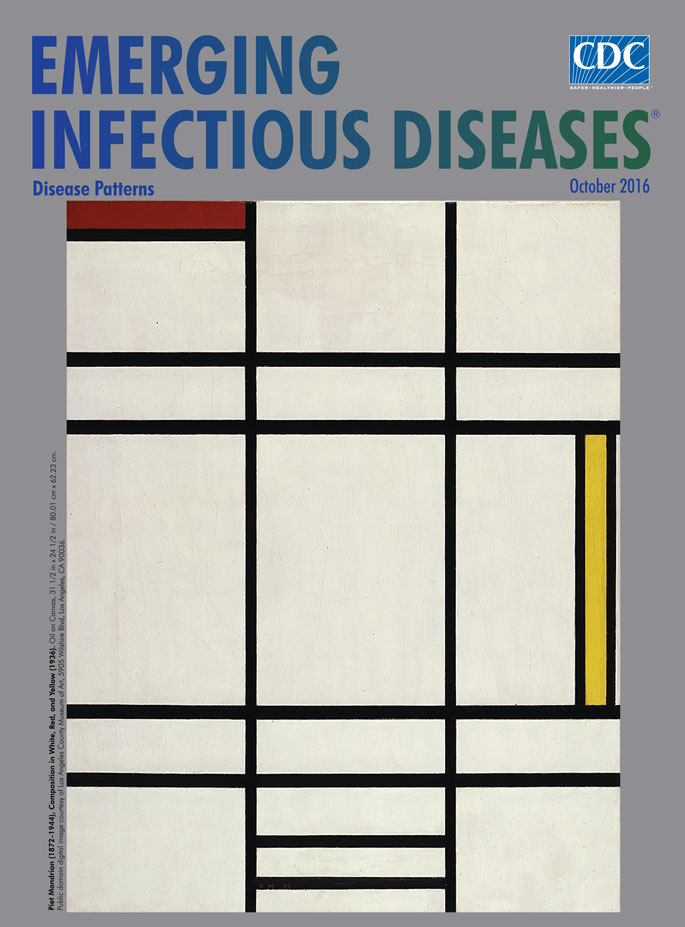
**Piet Mondrian (1872–1944), Composition in White, Red, and Yellow (1936). Oil on canvas, 31 1/2 in × 24 1/2 in /80.01 cm × 62.23 cm.** Public domain digital image courtesy of Los Angeles County Museum of Art, 5905 Wilshire Boulevard, Los Angeles, CA 90036.

In his “Composition in White, Red, and Yellow,” Dutch artist Piet Mondrian illustrated geometric abstraction. He accomplished this effect via two primary-colored units embedded in a grid of vertical and horizontal black lines that draw viewers to a flat-structured polished web. Mondrian distilled direction to perpendicular columns and rows, color to bright red and yellow and neutral white and black, shape to squares and rectangles, and form to intersecting lines and outlined units. He methodically pared down form to indispensable lines and stacked rich colors in a few blocks. Influenced by analytical cubism, which is renowned for breaking objects and images into components, Mondrian pioneered reductive form and color, through an abbreviated pictorial vernacular. In creating this seemingly simplified painting he championed a distinctive relationship between color and blocks, rendering asymmetric balance and harmony.

Black, bordering pigmented zones, creates a maze reflecting mathematical accuracy and distinct complexity. Moving a single line or changing one color might disrupt subtle accord and structural delineations. The painting portrays minimal, yet essential, information through blatant intersections, geometric shapes, and interlocking planes. While thick black lines separate blocks, the arrangement and colors radiate energy that is both kinetic and serene, stimulating and restful, dynamic and static. Mondrian imparted a delicate balance between unequal and equivalent counterparts by using indispensable and contrasting positive and negative energies of solid and void, horizontal and vertical.

Similar to the balance in Mondrian’s painting, corresponding energies of time and space, art and science, and medicine and public health create momentum and energy in our compartmentalized and integrated personal and professional experiences. As public health specialists, we analyze microscopic images as well as gigantic amounts of clinical, public health, and geospatial data to develop interventions that enhance individual and population health. Through intricate scientific and geostatistical methods, scientists and healthcare providers assess relationships between risk factors and disease and between behaviors and well-being. We frame prevention and control of health conditions—infectious or chronic, environmental or hazardous—in units and blocks of person, place, and time.

By pioneering geoanalysis of data and using a dot map, Dr. John Snow traced the 1854 cholera outbreak in Soho, London, England, to a public water pump on Broad Street (now Broadwick Street). Disabling a well pump by removing its handle, a reductive and a seemingly simple public health intervention, credited Snow with ending an outbreak, providing advocacy for public health changes in London water and waste systems, and contributing to modern epidemiology. Then and now, visual representations of public health data, including graphs and maps, help in understanding disease causation by showing locations of cases and highlighting similarities or differences between blocks, regions, and countries with and without infection. The web of disease causation, a core public health concept, pinpoints theories on population patterns of health and disease and embodies multiple and complex factors influencing disease dynamics and differentials between and within populations.

Well into the twenty-second century, public health professionals continue to track infection, direct prevention, advance treatment, and predict trends by culling information on disease transmission. By scrutinizing mazes and grids of public health data and leveraging digital technologies, we provide insights into causes and manifestations of disease, reduce data to strategies crucial for prevention and treatment, and strive to translate science into public health. Through distilling complex epidemiologic, molecular, and geospatial, data we address also disease syndemics–two or more conditions that interact synergistically and increase morbidity and mortality. Through the media, the public receives crucial information and graphics about affected populations and locations and about health conditions that are physiologically, socially, or clinically linked.

Geospatial data, virtual grid meta-databases, grid computing concepts, spatial analytical methods, visualization or data-display techniques, and color-coded geographic visualizations: these all enhance our understanding of public health threats and facilitate control of outbreaks, endemic diseases, epidemics, and pandemics. Such methods inform research and programs on the effectiveness of vaccination programs, whole-genome sequencing analysis, and cluster detection of infections and diseases. Similar to how artists craft and reveal their expertise in color, form, and perspective—in cubist and abstract art—public health professionals leverage their expertise in epidemiology, statistics, and communication and in behavioral, social, and laboratory sciences to integrate clinical care and public health. Mondrian’s network of black lines and colored blocks focuses on essence, equilibrates white-painted blocks with primary colored blocks, enunciates focal and peripheral information, and juxtaposes structural and dynamic equilibrium. We perceive similar reductive and essential processes and pictorials in public health. Art and science balance our health and enhance our lives.
